# Chronic Pulmonary Disease Caused by *Tsukamurella toyonakaense*

**DOI:** 10.3201/eid2807.212320

**Published:** 2022-07

**Authors:** Tomoki Kuge, Kiyoharu Fukushima, Yuki Matsumoto, Haruko Saito, Yuko Abe, Eri Akiba, Kako Haduki, Tadayoshi Nitta, Akira Kawano, Michio Tanaka, Yumi Hattori, Takahiro Kawasaki, Takanori Matsuki, Takayuki Shiroyama, Daisuke Motooka, Kazuyuki Tsujino, Keisuke Miki, Masahide Mori, Seigo Kitada, Shota Nakamura, Tetsuya Iida, Atsushi Kumanogoh, Hiroshi Kida

**Affiliations:** Osaka University, Osaka, Japan (T. Kuge, K. Fukushima, Y. Matsumoto, Y. Abe, M. Tanaka, Y. Hattori, T. Shiroyama, D. Motooka, S. Nakamura, T. Iida, A. Kumanogoh);; Toneyama Medical Center, Osaka (K. Fukushima, H. Saito, E. Akiba, K. Haduki, T. Nitta, A. Kawano, T. Kawasaki, T. Matsuki, K. Tsujino, K. Miki, M. Mori, H. Kida);; Kitada Respiratory Clinic, Osaka (S. Kitada)

**Keywords:** chronic pulmonary disease, nontuberculous mycobacteria, Tsukamurella toyonakaenses, Tsukamurella paurometabola, bacteria, rapid-growing mycobacteria, tuberculosis and other mycobacteria, antimicrobial resistance, Japan

## Abstract

Unidentified *Mycobacterium* species are sometimes detected in respiratory specimens. We identified a novel *Tsukamurella* species (*Tsukamurella* sp. TY48, RIMD 2001001, CIP 111916^T^), *Tsukamurella toyonakaense*, from a patient given a misdiagnosis of nontuberculous mycobacterial pulmonary disease caused by unidentified mycobacteria. Genomic identification of this *Tsukamurella* species helped clarify its clinical characteristics and epidemiology.

In clinical practice, unidentified *Mycobacterium* species are sometimes detected in respiratory specimens. Few *Mycobacterium* species can be identified by using methods available in clinical practice, although there are ≈200 species of nontuberculous mycobacteria (NTM) (*1*). We reported a case of pulmonary disease caused by a novel mycobacteria species identified by using multilocus sequence typing (MLST) and whole-genome sequencing (WGS) (*2*). 

## The Study

We investigated the epidemiology of unidentified pathogenic mycobacteria by using TRCReady MTB and MAC (Tosoh Bioscience, https://www.tosohbioscience.com), AccuProbe (Gen-Probe Inc., https://www.gen-probe.com), COBAS AMPLICOR (Roche Diagnostics, https://www.roche.com), and a DNA–DNA hybridization assay (Kyokuto Pharmaceutical Industrial, https://www.kyokutoseiyaku.co.jp). WGS analysis of preserved unidentified mycobacteria culture isolates was approved by the institutional research ethics board (TNH2019063–2). The requirement for informed consent was waived because of the retrospective nature of the analysis. The opt-out recruitment method was applied to provide an opportunity for all patients to decline participation. Results of WGS analysis of TY48 were deposited in BioProject (accession no. PRJDB10620) and BioSample (accession no. SAMD00250050).

We performed MLST and WGS of culture isolates from 8 patients given diagnoses of NTM pulmonary disease caused by unidentified mycobacteria. We identified *Mycobacterium shimoidei*, *M. shinjukuense*, *M. paragordonae*, *M. heckeshornense*, *M. lentiflavum* (3 isolates), and a novel *Tsukamurella* species (*Tsukamurella* sp. TY48, RIMD 2001001, CIP 111916^T^).

**Figure 1:**
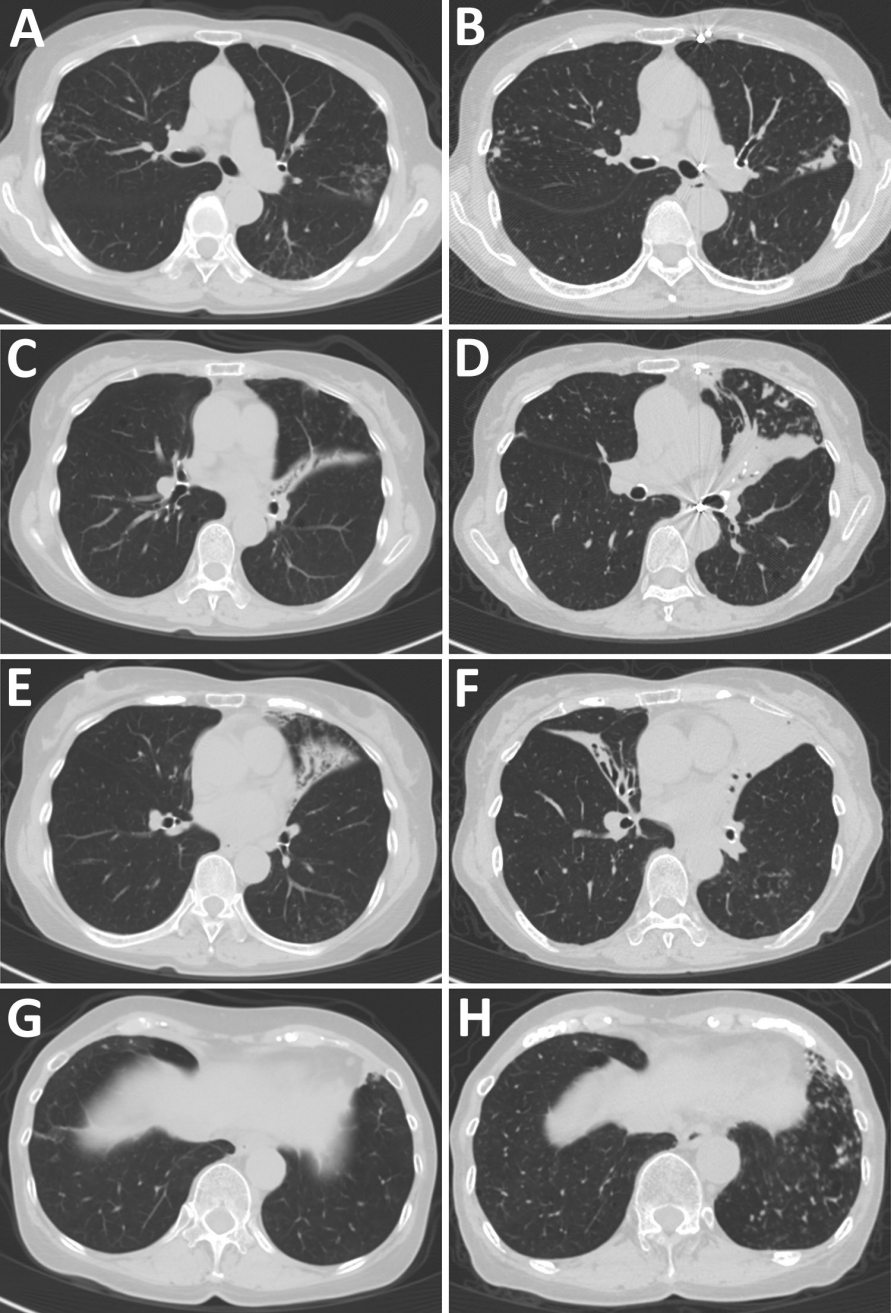
Comparison of chest computed tomography findings over time for patient who had chronic pulmonary disease caused by *Tsukamurella toyonakaense*. Findings are shown from before *Tsukamurella* species was detected (A, C, E, and G) and 6 years later (B, D, F, and H). A and B show that nodules in right segment 2 and left segment 6 were unchanged. C and D show that bronchiectasis in lingula had progressed. E and F show that bronchiectasis newly appeared in the middle lobe. G and H show that nodules newly appeared in left segments 8–10.

The patient with *Tsukamurella* infection was an 82-year-old woman who had received a diagnosis of NTM pulmonary disease 23 years earlier. Then a 59-year-old previously healthy woman, she was referred to our hospital because of abnormal chest radiographic findings. Although she had no symptoms, chest computed tomography findings showed centrilobular nodules and bronchiectasis. During follow-up, a cough and occasional hemoptysis developed. *M. chelonae* was repeatedly identified from her sputum. We started airway clearance therapy with erythromycin and expectorants. After 2 years of treatment, the *M. chelonae* disappeared from her sputum. However, her symptoms and radiologic findings slowly but steadily progressed (Figure 1), and rapidly growing acid-fast bacilli were repeatedly detected in her sputum for 8 years. The culture isolates were Ziehl-Neelsen stain positive. However, the species/subspecies could not be identified by using conventional methods. Therefore, she was given a diagnosis of NTM pulmonary disease caused by unidentified mycobacteria.

We continued erythromycin treatment for >20 years on the basis of evidence regarding successful treatment of NTM pulmonary disease with erythromycin (*3*). However, her symptoms and radiologic findings of lung destruction and structural alterations slowly but steadily progressed.

Because of this progression, we performed WGS by using a MinION Sequencer and Flow Cell R94 (Oxford Nanopore Technologies, https://nanoporetech.com). We extracted genomic DNA from cultured isolates by using a NucleoSpin Microbial DNA Kit (Takara Bio, https://www.takarabio.com) and prepared a library by using the Rapid Barcoding Kit (Oxford Nanopore Technologies). Using MinION raw sequencing reads, we performed MLST analysis on the 184-gene accessory genome with mlstverse software (https://www.multiverse.io) as reported (*1*). The unidentified mycobacterium was presumed to be *M. fallax* (MLST score 0.083). However, the low MLST score prompted a deeper analysis of the bacterial genome. 

**Figure 2:**
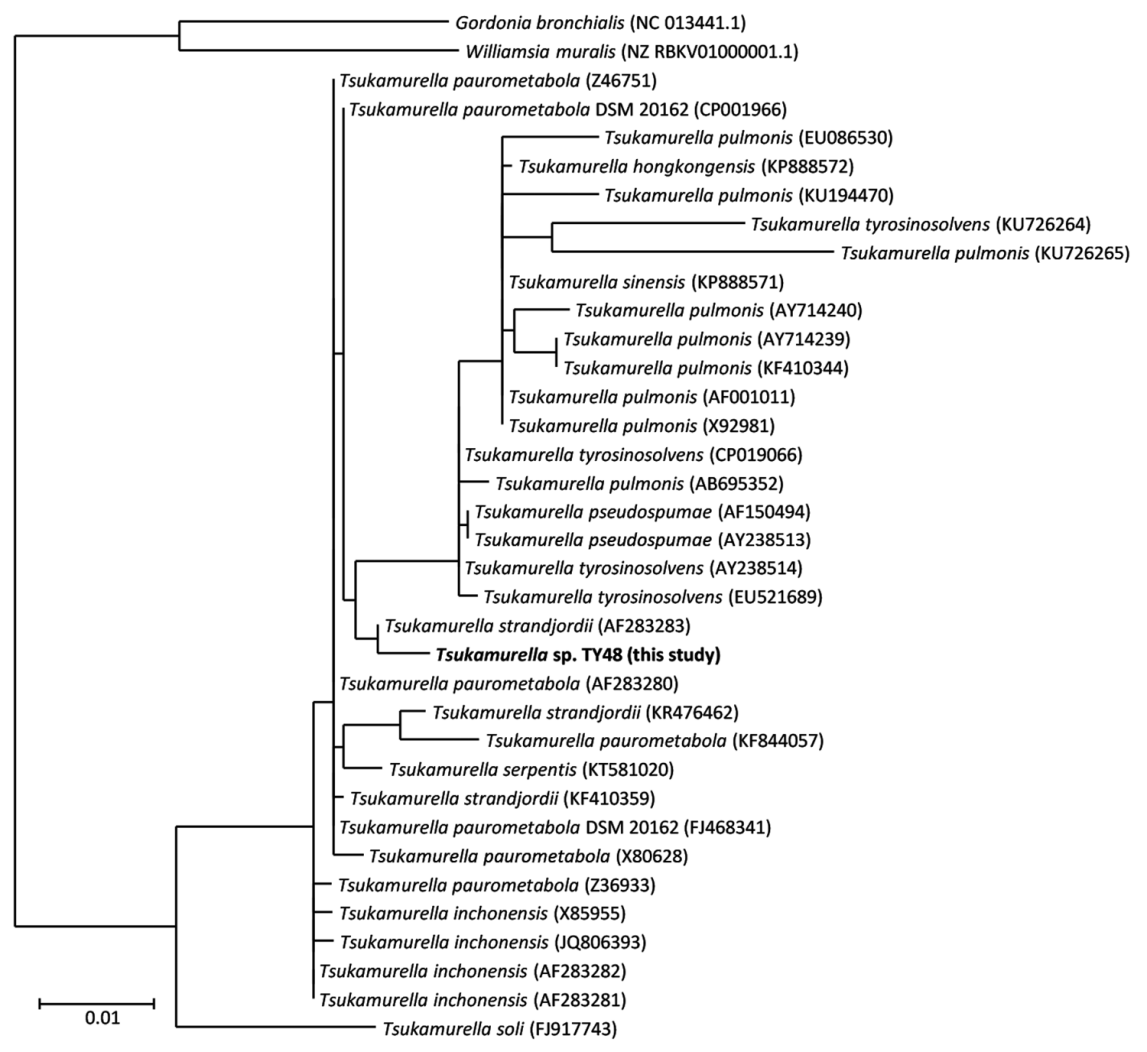
Maximum-likelihood phylogenetic tree constructed by using 16S rRNA sequences of *Tsukamurella* spp. and other bacterial species. Bold indicates strain isolated in this study. Reference sequences were obtained from SILVA database (*4*) release 138 as small subunit reference nonredundant 99 sequences, which showed >98.7% identity with strain TY48. GenBank accession numbers are provided for reference sequences. Scale bar indicates nucleotide substitutions per site.

We conducted a 16S rRNA analysis by performing a homology search using blastn (https://blast.ncbi.nlm.nih.gov) and compared our data with that in the SILVA rRNA database (*4*). The phylogenetic tree constructed using full-length 16S rRNA genes showed that strain TY48 was closely related to other *Tsukamurella* species (>98.7%), whereas its homology to 2 type species belonging to the related bacteria *Gordonia bronchialis* and *Williamsia muralis* was only 94.0% (Figure 2). We next determined the complete genome sequence of TY48 as reported (*1*) and performed WGS by using MinION and HiSeq 2500 instruments (Illumina, https://www.illumina.com). We performed genome assembly for strain TY48 by using flye (https://www.flye.com) for long reads obtained from MinION and corrected sequencing error by using pilon (https://bio.tools/pilon).

A comparison of the TY48 genome sequence with those of other *Tsukamurella* species indicated that the nearest related species was *T. paurometabola* (average nucleotide identity of 86.2%) (Table 1). This finding suggested that *Tsukamurella* sp. TY48 (RIMD 2001001; CIP 111916^T^) was a novel *Tsukamurella* species.

**Table 1:** Eight species of *Tsukamurella* used for calculation of average nucleotide identity*

Species	Strain	Reference sequence accession no.	Reference sequence category
*T. paurometabola*	DSM20162^T^	GCF_000092225.1	Representative genome
*T. tyrosinosolvens*	NCTC13231^T^	GCF_900637875.1	Representative genome
*T. pulmonis*	CCUG3572^T^	GCF_001575165.1	Representative genome
*T. sputi*	HKU70^T^	GCF_007858445.1	Representative genome
*T. conjunctivitidis*	HKU72^T^	GCF_007858475.1	Representative genome
*T. asaccharolytica*	HKU71^T^	GCF_007858435.1	Representative genome
*T. spumae*	DSM44113^T^	GCF_012396015.1	NA
*T. pseudospumae*	JCM15929	GCF_001575195.1	Representative genome

*NA, not available.

We performed antimicrobial drug susceptibility tests for rapidly growing mycobacteria by using the broth microdilution method in accordance with Clinical and Laboratory Standards Institute M24-A2 guidelines (*5*). We transferred the culture to Middlebrook 7H9 broth and vortexed. We adjusted the culture medium to a 0.5 McFarland standard with sterile distilled water; we then added 60 μL of the 0.5 McFarland suspension to a Cation-Adjusted Mueller-Hinton Broth (Kyokuto Pharmaceutical Industrial Co. Ltd., https://www.kyokutoseiyaku.co.jp) and dispensed 100 μL of this solution into each well of the panel. After confirming adequate growth of the control over a 3-day incubation in a standard atmosphere at 30°C, we determined the MICs (μg/mL) for 15 drugs: clarithromycin, 0.25; azithromycin, <0.25; cefoxitin, <8; imipenem, <0.5; meropenem, <0.5; faropenem, <1; amikacin, <1; tobramycin, 2; minocycline, <0.25; doxycycline, <1; linezolid, <4; moxifloxacin, <0.25; ciprofloxacin <0.5; levofloxacin, <0.5; and trimethoprim/sulfamethoxazole, <2/38. *Tsukamurella* sp. TY48 was sensitive to all 15 drugs.

We renamed TY48 as *T. toyonakaense* after the location of its discovery, Toyonaka, Japan. *T. toyonakaense* is an aerobic, nonmotile, gram-positive rod that grows at 30°C and 37°C, but not at 42°C, and produces catalase. After a 72-h incubation at 30°C on 7H11 agar, it forms white and creamy, rough, nonpigmented colonies (10 mm in diameter). According to the API 50 CH system (bioMérieux, https://www.biomerieux.com), this bacterium can assimilate fructose, glucose, starch, sucrose, and trehalose but not arabinose, mannitol, mannose, or xylose.

After diagnosis, we attempted combination drug therapy with clarithromycin (200 mg/d) and ethambutol (250 mg/d). The patient refused continuation of treatment after 2 weeks because of antimicrobial drug–induced fatigue. We then resumed treatment with erythromycin. Her symptoms and radiologic findings are slowly improving (Table 2; Appendix).

**Table 2:** Characteristics of 10 patients who had *Tukamurella* pulmonary disease*

Report author, year	Patient, age, y/sex/country	Medical history	Signs/symptoms	Imaging findings	Initial diagnosis of infection	Identified pathogen/ diagnostic method	Initial treatment; clinical response	Subsequent treatment
Tsukamura et al., 1982	50/M/Japan	None	Fever, cough for 1 week	Cavity, pleural effusion	*Mycobacterium tuberculosis*	*Gemmatimonas aurantiaca*/biochemical tests	INH, RFP, STM for 2 months; improved	NA
Alcaide et al., 2004	55/M/USA	Cutaneous T-cell lymphoma, AIDS	Fever, cough, fatigue, for 2 weeks	Cavity, bilateral infiltrates	*Mycobacterium*	*Tsukamurella* sp./biochemical tests	CPF, RFB for 12 weeks; cured	NA
Perez et al., 2008	71/M/USA	None	Fever, cough, hemoptysis for 3 months	Cavitary mass	*Mycobacterium*	*T. pulmonis*/biochemical tests	RFB, LVF for 6 months; cured	NA
Maalouf et al., 2009	76/M/USA	Non-Hodgkin lymphoma, COPD	Fever, cough, fatigue, for 4 days	Bilateral infiltrates	*Streptococcus pneumoniae*	*T. pulmonis*/biochemical tests, 16S rRNA sequencing	MEP, VCM for 10 days; symptoms decreased	CPFX and RFP for 4 months; improved
Menard et al., 2009	54/M/France	Lung transplant 4 years ago	Cough for 10 days	NR	Gram-positive bacilli	*T. tyrosinosolvens*/16S rRNA and hsp65 sequencing	IPM, TOB; symptoms decreased	NA
Inchingolo et al., 2010	76/F/Italy	COPD, diabetes mellitus, bilateral glaucoma.	Altered state of consciousness, dyspnea for 2 days	Ground-glass opacities, infiltrates, pleural effusion	*Staphylococcus epidermidis*	*T. pulmonis*/16S rRNA sequencing	AMP/CVA, AZM→AMP/CVA, CPF; symptoms decreased	CPFX for 10 days, cured
Mehta et al., 2011	79/M/USA	Coronary artery disease, atrial fibrillation	Fever, cough, bloody tinge for 10 days	Infiltrate	*Mycobacterium* sp.	*Tsukamurella* sp./HPLC	First-line CTR, AZM/ NE; second-line INH, RFP, EMB, PZA, AZM/NE	CPFX and AZM, improved
Chen et al., 2016	75/M/Taiwan	Diabetes mellitus, COPD	Fever, cough, general malaise for 1 week	Infiltrate	*Nocardia* sp.	*T. tyrosinosolvens*/biochemical tests, 16S rRNA sequencing	First- line CTR, AZM for 1 week, treatment failure; second-line IPM for 3 weeks, STM for 4 weeks; condition improved	INH; EB; and RFP for 12 months, improved
Yang et al., 2017	24/F/Unknown	None	Fever, cough, hemoptysis	Cavity, infiltrated mass	*Mycobacterium tuberculosis*	*T. paurametabora*/16S rRNA sequencing	First-line standard antituberculosis treatment, symptoms improved; second-line intensive antituberculosis therapy for 20 days; treatment failure	LNZ for 3 weeks, improved
This study	81/F/Japan	NTM-PD	Cough, hemoptysis for years	Bilateral centrilobular nodules, bronchiectasis	Rapidly growing mycobacterium	*Tsukamurella* sp. nov./WGS	ERY 20 years, slow progress	NA

*Full reference lists for *Tukamurella* pulmonary disease reported cases are shown in Appendix . AMP/CVA, amoxicillin/clavulanate; AZM, azithromycin; CPF, ciprofloxacin; CTR, ceftriaxone; EMB; ethambutol; ERY, erythromycin; HPLC, high-performance liquid chromatography; hsp, heat-shock protein; INH, isoniazid; IPM, imipenem; LNZ, linezolid; LVF, levofloxacin; MEP, meropenem; NA, not available; NE, not evaluated; NTM-TB, nontuberculous mycobacteria tuberculosis; NR, not reported; PZA, pyrazinamide; RFB, rifabutin; STM, streptomycin; SMX/TMP, sulfamethoxazole/trimethoprim; TOB, tobramycin; VCM, vancomycin.; WGS, whole-genome sequencing.

## Conclusions

*Tsukamurella* species are aerobic, gram-positive, partially acid-fast, and nonmotile bacilli that can cause opportunistic infections, including pulmonary disease (*6*). Sixteen species of *Tsukamurella* have been classified (*7*). Only 9 pulmonary disease cases have been reported (*8**,**9*) (Table 2). 

The prevalence of *Tsukamurella* pulmonary disease is probably underestimated. The genus *Tsukamurella* is often misidentified as related genera because it is difficult to identify in most clinical microbiology laboratories (*10*). Because of its partially acid-fast bacilli and cavitary shadow in radiologic examination, *Tsukamurella* pulmonary disease is often confused with *Mycobacterium* infection and often treated with antituberculous drugs (*9*). Yu et al. genotyped specimens from 101 NTM pulmonary disease patients by using 16S rRNA and 16S‒23S rRNA internal transcribed spacer sequences and detected *Tsukamurella* species in ≈1% of the specimens (*11*). If one considers the prevalence of NTM pulmonary disease, the actual prevalence of *Tsukamurella* pulmonary disease is probably much higher than the 9 reported cases.

*Tsukamurella* commonly causes acute onset pneumonia with cavity and consolidation (Table 2) and fever, coughing, sputum, fatigue, and hemoptysis. Although appropriate drugs and treatment durations are unknown, combination medications of >2 drugs, including rifampin or quinolone, are widely used and presumed effective on the basis of case reports (*6**,**8**,**12**–**14*). These reports also indicated a good prognosis for *Tsukamurella* pulmonary disease (*8**,**12**,**13*). No relapses were reported, in contrast to NTM pulmonary disease. Although the Clinical and Laboratory Standards Institute has proposed breakpoints for aerobic actinomycetes (*5*), no definitive drug breakpoints for *Tsukamurella* spp. have been established. However, the strain we identified showed extensive antimicrobial drug susceptibility.

Because a clinically applicable identification technique is not available, *Tsukamurella* infections are probably underestimated and more prevalent than has been recognized. Misidentification as related genera, especially *Mycobacterium*, results in missed opportunities to properly treat *Tsukamurella* infections. Use of genomic sequencing to identify *Tsukamurella* species and more cases of *Tsukamurella* infections will help identify clinical characteristics and clarify epidemiology of *Tsukamurella* pulmonary disease.

Appendix Additional information on chronic pulmonary disease caused by *Tsukamurella toyonakaense*.
