# Supplementary material for: Chronic Pulmonary Disease Caused by Tsukamurella toyonakaense
Source: Emerg Infect Dis. 2022 Jul;28(7):1437–41. doi: 10.3201/eid2807.212320 (PMC9239891; doi:10.3201/eid2807.212320)
Supplement: Appendix — Additional information on chronic pulmonary disease caused by Tsukamurella toyonakaense. [file 21-2320-Techapp-s1.pdf]

Article DOI: https://doi.org/10.3201/eid2807.212320

Chronic Pulmonary Disease Caused by *Tsukamurella toyonakaense*

Appendix

References

1. Tsukamura M, Kawakami K. Lung infection caused by *Gordona aurantiaca* (*Rhodococcus aurantiacus*). J Clin Microbiol. 1982;16:604–7. PubMed https://doi.org/10.1128/jcm.16.4.604-607.1982

2. Alcaide ML, Espinoza L, Abbo L. Cavitary pneumonia secondary to *Tsukamurella* in an AIDS patient. First case and a review of the literature. J Infect. 2004;49:17–9. PubMed https://doi.org/10.1016/S0163-4453(03)00139-7

3. Perez VA, Swigris J, Ruoss SJ. Coexistence of primary adenocarcinoma of the lung and *Tsukamurella infection*: a case report and review of the literature. J Med Case Reports. 2008;2:207. PubMed https://doi.org/10.1186/1752-1947-2-207

4. Maalouf R, Mierau SB, Moore TA, Kaul A. First case report of community-acquired pneumonia due to *Tsukamurella pulmonis*. Ann Intern Med. 2009;150:147–8. PubMed https://doi.org/10.7326/0003-4819-150-2-200901200-00022

5. Ménard A, Degrange S, Peuchant O, Nguyen TD, Dromer C, Maugein J. *Tsukamurella tyrosinosolvens*: an unusual case report of bacteremic pneumonia after lung transplantation. Ann Clin Microbiol Antimicrob. 2009;8:30. PubMed https://doi.org/10.1186/1476-0711-8-30

6. Inchingolo R, Nardi I, Chiappini F, Macis G, Ardito F, Sali M, et al. First case of *Tsukamurella pulmonis* infection in an immunocompetent patient. Respir Med CME. 2010;3:23–5. https://doi.org/10.1016/j.rmedc.2009.02.004

7. Mehta YB, Goswami R, Bhanot N, Mehta Z, Simonelli P. *Tsukamurella* infection: a rare cause of community-acquired pneumonia. Am J Med Sci. 2011;341:500–3. PubMed https://doi.org/10.1097/MAJ.0b013e3182129d02

8. Chen CH, Lee CT, Chang TC. *Tsukamurella tyrosinosolvens* bacteremia with coinfection of *Myobacterium bovis*: a case report and literature review. Springerplus. 2016;5:52033.6. https://doi.org/10.1186/s40064-016-3707-y PMID: 27995010

9. Yang L, Cao Y, Dan Z, Wang Z, Wang X. Community-acquired *Tsukamurella* pneumonia in a young immunocompetent adult: a case misdiagnosed as pulmonary tuberculosis and literature review. Postgrad Med. 2017;129:563–6. PubMed https://doi.org/10.1080/00325481.2017.1344513
